# Research on nano H_2_/O_2_ bubble generating mechanism and characteristics

**DOI:** 10.3389/fchem.2022.919114

**Published:** 2022-09-05

**Authors:** Chia-Lung Kuo, Chin-Ta Chen, Chao-Ching Ho

**Affiliations:** ^1^ National Yunlin University of Science and Technology, Douliu, Taiwan; ^2^ National Taipei University of Technology, Taipei, Taiwan

**Keywords:** nano bubble, fuel liquid, nano H_2_/O_2_ bubble generating, hollow electrode, bubble generating mechanism

## Abstract

In this research, electrolysis water is used to produce hydrogen and oxygen for carrying out the vertical cutting through high-speed water in order that the bubbles will be refined for generating the nano H_2_/O_2_ bubble liquid. In the meantime, a Nanobubble Generator is developed to verify the basic characteristics of the produced nano H_2_/O_2_ bubbles. Its purpose is to identify the maximum concentration of bubbles in the nano H_2_/O_2_ bubble liquid, the bubble production efficiency and bubble electrification characteristics as well as the effect of reducing the pipe flow friction resistance together with the characteristics of nanobubbles containing varied gases. By verifying the nano H_2_/O_2_ bubbles, it is hoped that the flowing rate of the hollow electrode can be elevated.

## Introduction

### Preface

Until now, microbubble technology has been developed for over 30 years in the global market. The microbubble liquid containing 50 μm or even smaller microbubbles is designed with many physical characteristics in terms of cleaning, sterilization effect, and electrification performance and it has now attracted the profound interest of major factories. In the meantime, it is also widely applied in the industrial, agricultural, medical, nurturing, and stock farming sectors. The viscosity of water (0.8 mPa-s) is 44.5 times that of air (18 μPas). When the nanobubble processing liquid is formed in the gas-mixed liquid, it can effectively reduce the friction resistance between fluid and flowing path (Jeung and Yong, 2020; Mayrhofer et al., 2020) and promote hydrate formation (Uchida et al., 2020; Wang et al., 2021). The nanobubble-related article is proposed by Yoshiaki Kodama and Akira Kakugawa et al., in 2000 (Kodama et al., 2000) where a circulation water tunnel specially designed for the microbubble experiment is used to conduct the microbubble testing. Such an experimental tunnel is provided with a longer testing zone in order to measure the durability of the microbubbles flowing along the path in reducing the friction on the skin. During the test, a skin friction transducer is used to measure the skin friction status and such transducer resembles the dynamometer provided with a 250 N/m^2^ full range. Through the microbubbles, the skin friction rate has been reduced by 40%. In the meantime, we also placed a straw in the tested liquid to measure the local porosity under the bubbling status. The test result suggested the local porosity near the wall is closely related to the skin friction resistance. In 2003, Masayoshi Takahashi and Taro Kawamura et al. (Takahashi et al., 2003) mixed 1% of tetrahydrofuran (THF) with the distilled water in the tank so that it will be combined with the microbubbles for testing how the microbubbles will become the hydrate. The result proved that the microbubble system is a very potential hydrate forming method due to the following two reasons: 1) Excellent gas dissolving capability; and 2) Preparation capability allowing much easier nucleation conditions for the hydrate. Further, in 2003, A. Fujiwara et al. (2003), proposed an effective technology allowing the bubbles to concentrate in the diffusing nozzle (Venturi). Through the bubble fission effect created by instant restoring of pressure in the nozzle, the microbubbles in diameter rated about 100 μm are produced. In 2017, scholars W. Bouhijanto et al.,(Wiratni et al., 2017) studied the feasibility of using a new type of aerator, i.e., the so-called Microbubble Generator (MBG) in freshwater fish aquaculture farm. Such MBG is operated according to Venturi theory allowing the water to circulate by flowing through a narrow channel where the air will be sucked into the device and pushed by the flowing water to produce the microbubbles. In 2005, Jiacai Lu, Arturo Fernández, and Gretar Tryggvason (Lu et al., 2005) proposed a direct value simulation method in which bigger-sized bubbles are injected into the inner wall of the “narrowest turbulent channel” in order to confirm the influence of the bubbles on the channel wall resistance. The front-tracking/finite-volume method is used to solve all of the flow rate scales, including the flow rate of bubbles and the surrounding areas and the bubble diameter is rated at 54 wall units. The result indicated that the flexible bubbles can pass through the current flow vortex and the wall resistance is significantly reduced. In 2005, Japanese scholar Masayoshi Takahashi (Takahashi, 2005) proposed a bubble generating mechanism with which the gas will be guided from the air intake automatically through the centrifugal force produced by the circulation of the water. After entering the Bubble Generating Mechanism, the gas will flow along the central shaft and the gas vortex is created. Through the strong shearing force and the circulation force of the water, the gas is separated into tiny bubbles at the outlet of such a mechanism. In the meantime, the Microscope System featured on 200–400 magnification, 640 × 480 image pixels, and 30 fps is used with the graphical figures for determining the relationship between the measured bubble diameter and the bubble rising speed. The result indicated that the estimated bubble diameter measuring error is less than 5%. The bubble rising speed is evaluated according to the distance when each individual bubble moves within a duration longer than 1 s. In 2007, Japanese scholar Takahashi (Takahashi et al., 2007) proposed that properties different from ordinary bubbles are present when bubbles are under a tiny status. It is not the size issue only because they are provided with different characteristics, and are called microbubbles and nanobubbles. In this regard, microbubbles are bubbles with a diameter rated at 50 μm or even smaller. When normal bubbles are rising and breaking apart quickly in the water and then disappear on the surface, they will be contracted in the water and then disappear in the long run. During the contraction process, the organic electrolytic ions are surrounding the microbubbles in a highly concentrated manner and then inhibited the gas dissolving effect in the bubbles. As a result, it allows the microbubbles to stay in a stabilized status for a longer time. When microbubbles disappear in the water, it is very important in producing free radicals and in serving as residual nanobubbles. Because free radicals are excellent in chemical substance decomposition, they are provided with lots of functions such as water treatment techniques. To effectively produce such types of nanobubbles, they should be produced in water containing a certain quantity of electrolyte so that they will be floating naturally. For another, the nanobubbles are tiny bubbles in diameter rated at 1 μm and even smaller and shall be present in the water for a certain period of time. In 2010, scholars Fermanda, Yumi, and Ushikubo et al. (2010) studied the presence and the stability of nanobubbles after generating the bubbles. In the meantime, they also applied pure oxygen to produce the bubbles and then test the bubbles with DLS (Dynamic Light Scattering) method 1 h later. The result suggested that the bubble existence time is related to the size of the bubble. After introducing the O_2_ microbubbles and the nanobubbles, the potential (ζ) measured in the water and the air bubbling measured in the water are between −45 mV and −34 kV, and between −20 mV and −17 mV, respectively. The research result indicated that it is highly possible that the nanobubbles can be present in the water for a longer period of time. The nanobubbles are stabilized because of the electrified liquid-gas interface (preventing the bubble conglomeration repulsive force) and the high concentration of gas dissolved in the water (maintaining a smaller concentration gradient between interface and main body). In 2012, scholars Khuntia, S. (Khuntia et al., 2012) et al. suggested that the bubble-producing method will affect the attributes of nanobubbles. There are four kinds of methods used to produce the fluid mechanics and they are fluid dynamics, acoustics, optics, and particle holes. When compared with other methods, the hydraulic cavity is a method lately developed and it has been confirmed as more cost-efficient and more efficient. The method used to produce the spiral fluid flow (MNBs) is a fluid dynamic hole method being widely applied. In 2020, Jeung Y. et al. (Jeung and Yong, 2020) employed electrochemical water splitting (EWS) to induce bubble drag reduction. In 2020 Mayrhofer L. et al. (Mayrhofer et al., 2020) also concluded that water splitting can lead to extremely low friction. Therefore, the further efficient use of energy is achieved by a lower coefficient of friction. Research findings indicate that nanobubbles and nanofluids are potentially advantageous as a coolant (Manigandan and Kumar., 2019) for photovoltaic fluid collector systems e.g., reducing the cell temperature and increasing the production of electricity (Anderson et al., 2022) and enhancing the system efficiency with the higher mass flow rate of the nanofluids (Sangeetha et al., 2021).

In view that viscosity is the basic physical property of the fluid, it can be regarded as the resistance resulting from the friction (internal friction) between molecules in the fluid. When the resistance is created under the effect of shearing force, the scale of such resistance will be proportional to the flowing velocity gradient of the fluid. In this case, the resulting viscosity coefficient is termed as the viscosity and it is the general characteristic of the fluid. During the research, a hollow electrode tube is used. When water is flowing through the slim tube, significant resistance is created that leads to serious energy loss when water is pushed out from the center channel with a high-pressure motor (250 bar). For this reason, the microbubble generating mechanism is developed for this research. In the meantime, the nanobubbles are applied to reduce the flowing channel friction resistance in order to confirm that it can improve the flow rate of the pipe flow.

### Microbubble device

#### Features of microbubbles

In the report prepared by Japan’s National Institute of Advanced Industrial Science and Technology (AIST) (Hideki, 2010), it pointed out that the features of nanobubbles can be classified into the following four types and they are bubble size, bubble concentration, bubble lifespan, and bubble electrification performance. Described below are the details of the aforesaid features:

Bubble size: The size of bubbles produced for this research is about 100 nm, which is smaller than the wavelength of visible light (390 nm–780 nm) and cannot be seen by naked eyes (high transparency).
Bubble concentration: The concentration is measured with a nanoparticle trace analyzer. In every single ml of nanobubble concentration produced by this research, it contains over one billion (10^9^) bubbles. When present in the water, the bubbles also reduce the liquid viscosity apart from increasing the oxygen content.
Bubble lifespan: As soon as the microbubbles (about 50 μm) are shrinking gradually, getting dissolved in the water, and then disappearing, the microbubbles (>50 μm) will absorb the gas dissolved in the water and get expanded gradually. After that, they will float on the water surface, get burst, and vanish. If every single “μm” of nanobubbles is less than 1 (several dozens of “nm” to several hundreds of “nm”), the volumetric size will remain unchanged and they can stay in the water for up to several months without bursting.
Bubble electrification performance: Normally bubbles (surface potential) are carrying negative charge (several dozens of “mV”) and they are related to the level of pH in the water. If the pH is less than 4, the potential is about +40mV; if the pH is higher than 4, then the potential will be ranging between 0 and −100 mV. The potential is mainly determined according to the concentration level of $"H^{+}$ and $OH^{-}$ “ that is adsorbed to the interface between bubbles and the water. Owing to such kind of electrification characteristics, the bubble conglomeration issue will not exist (scattering feature) that they can remain in the water for a longer period of time without getting conglomerated, floated, and dissipated.
Bubble gas: During the experiment, different bubble generating mechanisms are applied to convert the gas in the bubbles from air to H_2_/O_2_ (existing in separated bubbles) and H_2_+O_2_ (mixed in the same bubbles). In this way, the optimized effect is achieved through the properties of different gases.

#### Water electrolysis nano H_2_/O_2_ bubble generating device

**FIGURE 1:**
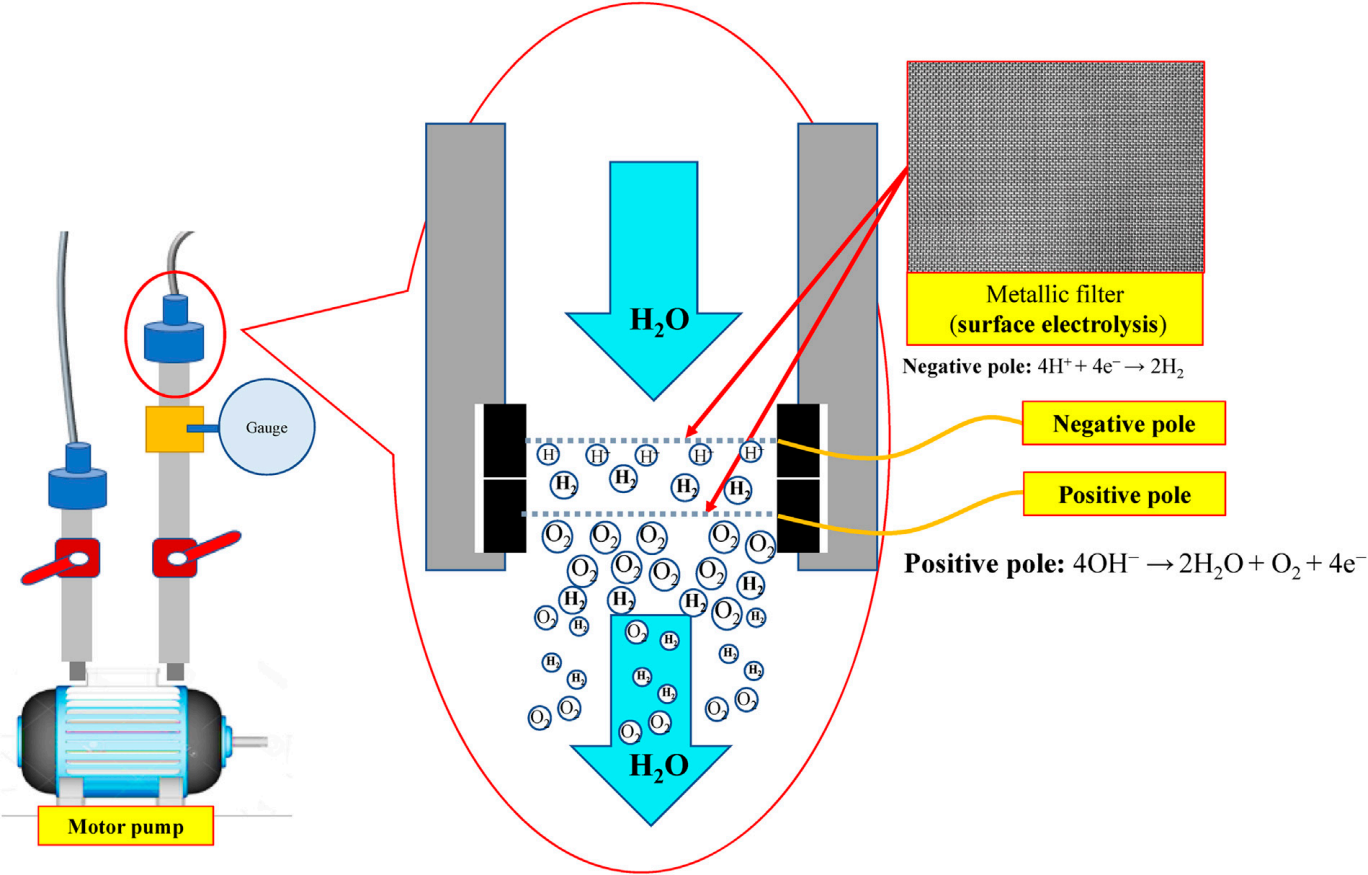
Structural system schematic diagram for electrolytic nano H_2_/O_2_ bubble generating device.

**FIGURE 2:**
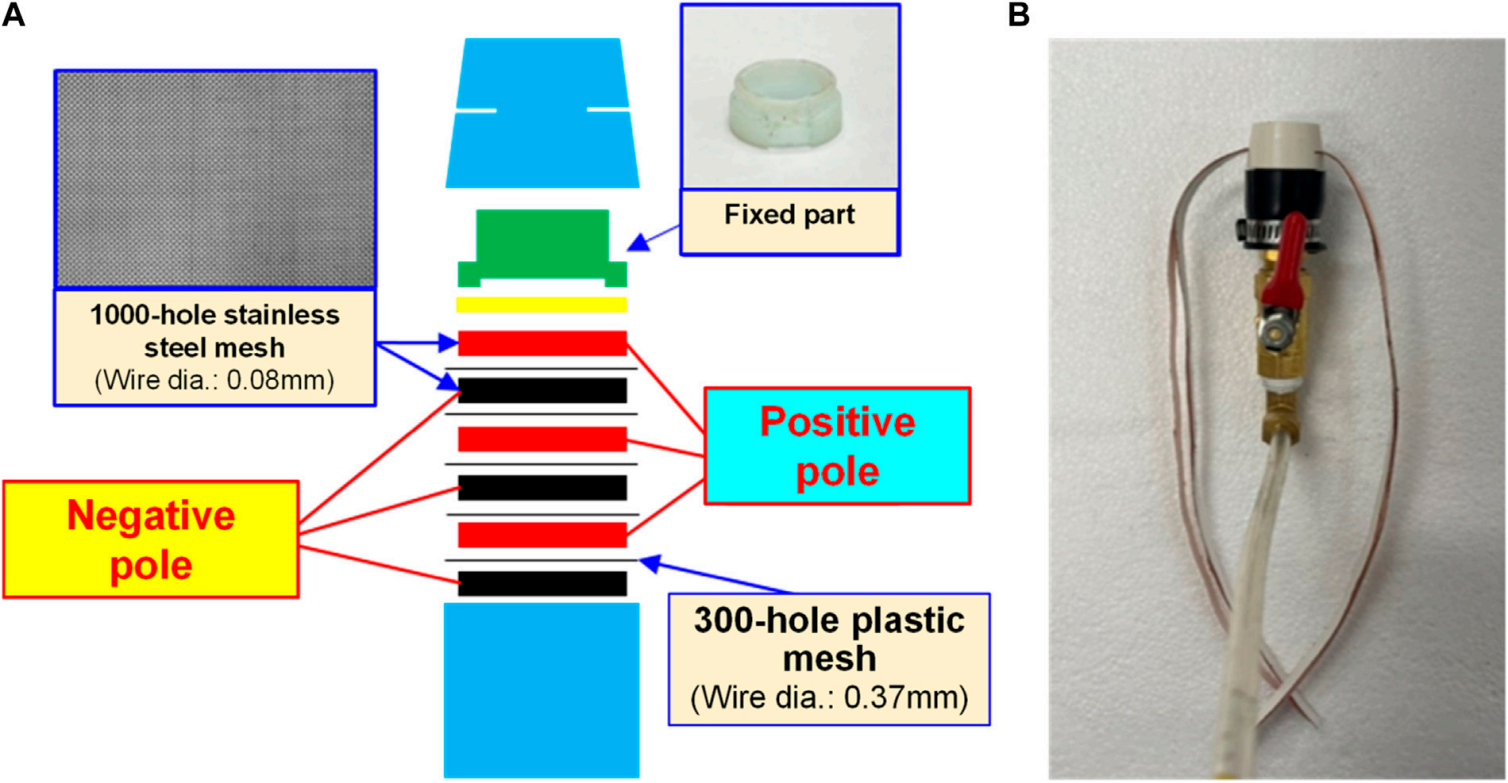
Electrolytic module **(A)** schematic diagram and **(B)** physical picture.

**FIGURE 3:**
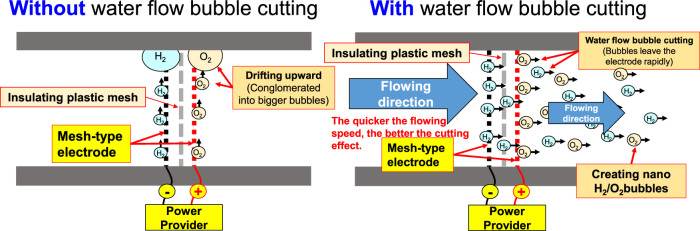
Water current bubble cutting schematic diagram for pipe flow.

Indicated in Figure 1 and Figure 2 is the theory of electrolytic nano H_2_/O_2_ bubble generating device for which, the RO (reverse osmosis) water (in electrolytic voltage about 6 V) or the deionized water (in electrolytic voltage about 15 V) is used. During the test, positive and negative charges are connected to the stainless steel mesh electrodes respectively. After energizing the electrode, the electrolytic effect is created to produce hydrogen (4H^+^ + 4e^−^ → 2H_*2*_) on the negative surface and oxygen (4OH^−^ → 2H_2_O + O_2_ + 4e^−^) on the positive surface respectively. During the entire process, the electrode is installed at the pump outlet or inlet. Being pushed by the water current produced by the pump, the gas flows quickly through the mesh-type electrode. When passing through the electrode surface, the hydrogen and the oxygen bubbles will be cut apart by the water current at their initial forming stage and then they will be brought away from the electrode surface in forming the nano H_2_/O_2_ bubbles (H_2_ and O_2_ existing in different bubbles). In this way, it will be difficult for them to form bigger bubbles, as per Figure 3. The sectional profile of the mesh-type electrode is configured in a round shape (in wire dia. rated at Ø0.08 mm). When the wire diameter gets smaller, the contacting angle between the same-size bubbles and the electrode becomes smaller and it will be easier for the bubbles to leave the electrode surface (because of the smaller adsorbing force). Described below is the analysis of the features of an electrolytic nano H_2_/O_2_ bubble generating device. 1) Higher electrolytic efficiency: With a very low current (0.01 A) (in voltage rated at 6–15 V), the gas required for producing the nanobubbles will be separated. Further, the bubbles produced on the negative and positive electrode surface will also be flushed by the high-speed water current immediately instead of staying on the electrode surface (forming the resistance). As such, pretty high electrolytic efficiency is achieved. 2) Simple in structure/lower in cost: Powerful nano H_2_/O_2_ bubble liquid can be produced simply with a compact structure and electrolytic mechanism. 3) Hydrogen/oxygen burning: Compared to 21% oxygen combusting effect used by air bubbles, the gas produced through the electrolytic process contains 100% combustible hydrogen that helps the burning of oxygen. 4) Wider application scope: By installing at the outlet end of the commercial pumps as well as the tooling machines such as fine hole/linear cutting discharge machining tools, it will be ready to produce the desired nano H_2_/O_2_ bubble liquid.

#### Bubble refining device for porous disc rotary cutting

**FIGURE 4:**
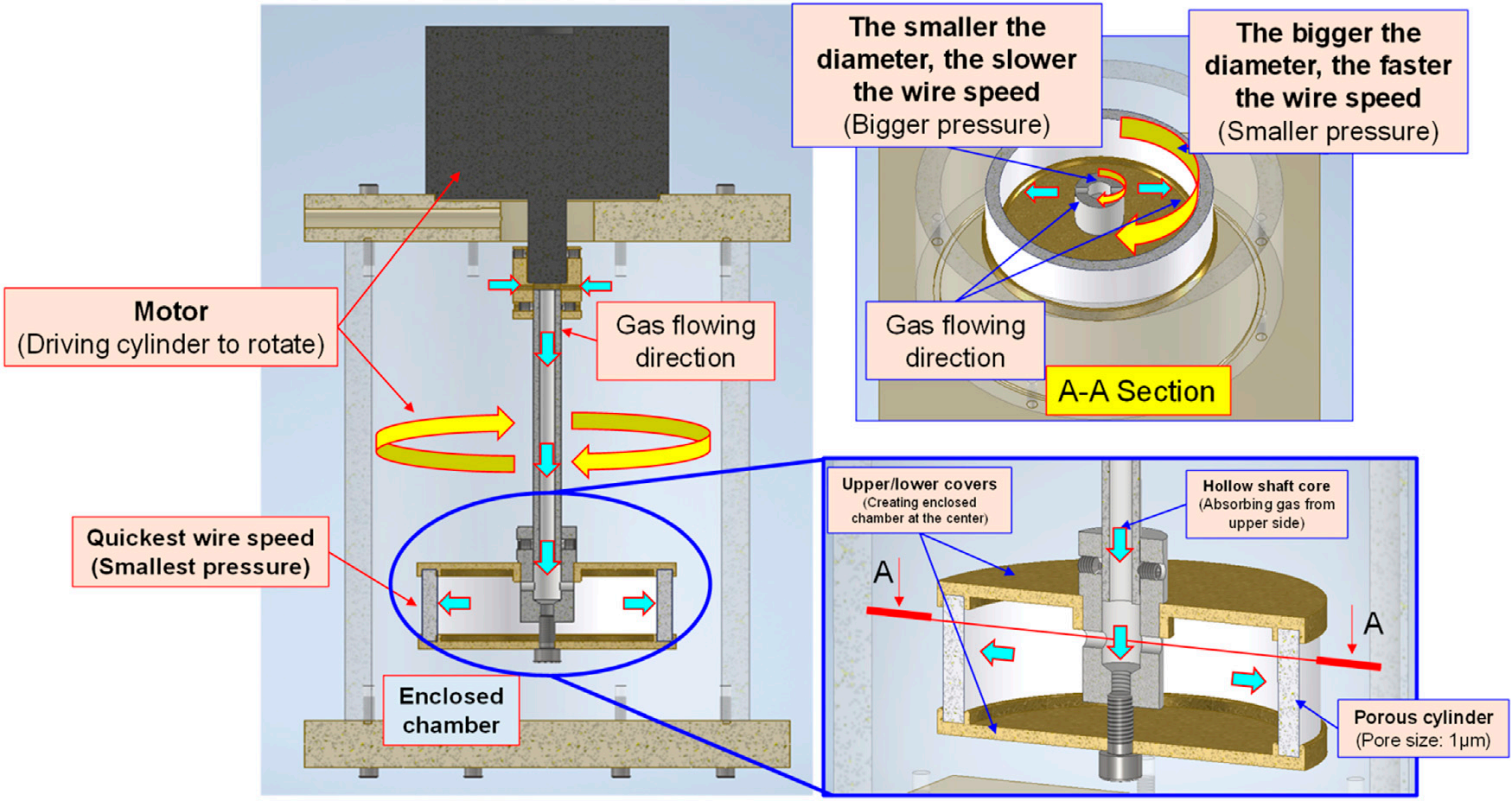
Schematic diagram and structural layout of bubble refining device for porous disc rotary cutting.

**FIGURE 5:**
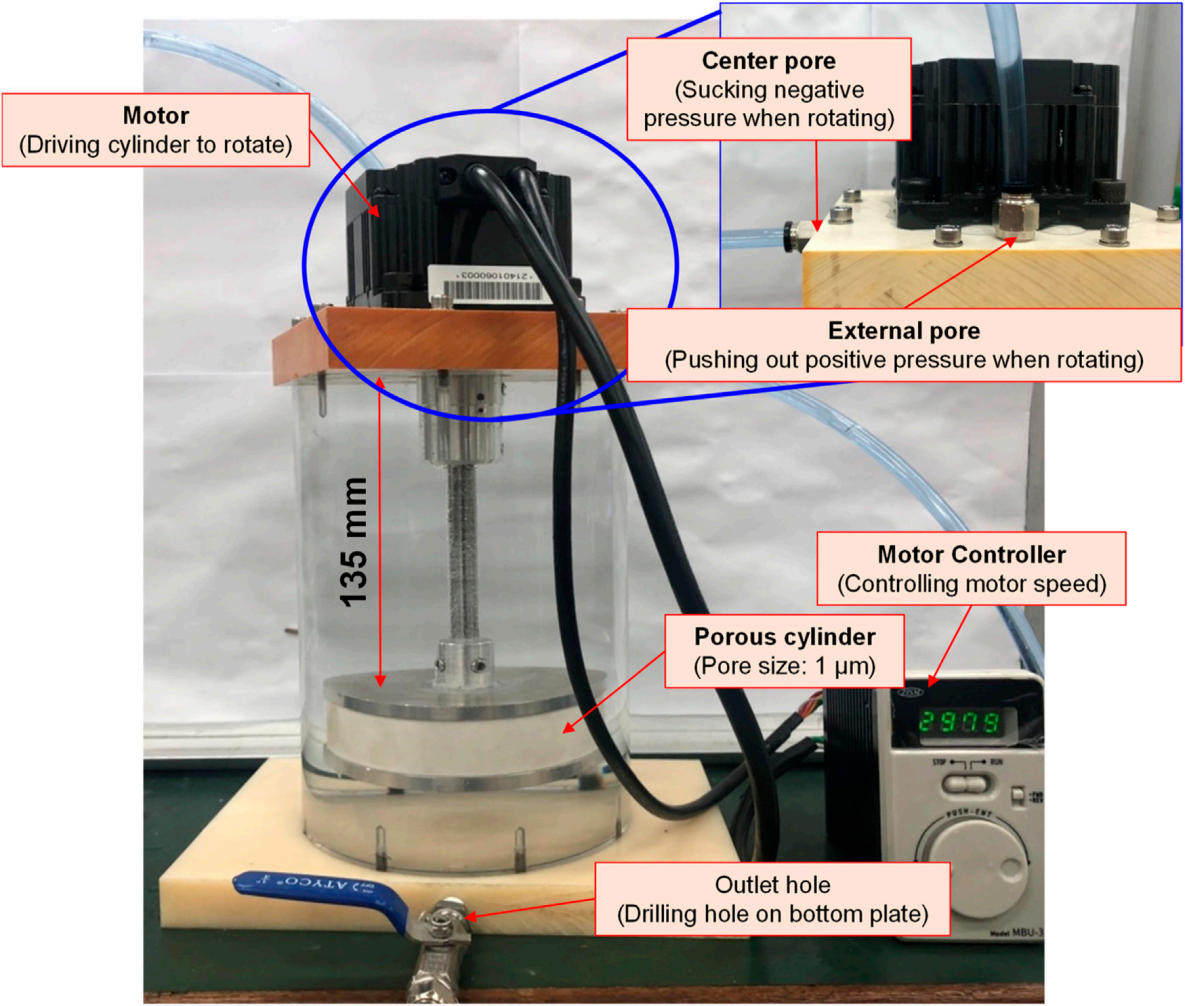
Physical layout of bubble refining device for porous disc rotary cutting.

**FIGURE 6:**
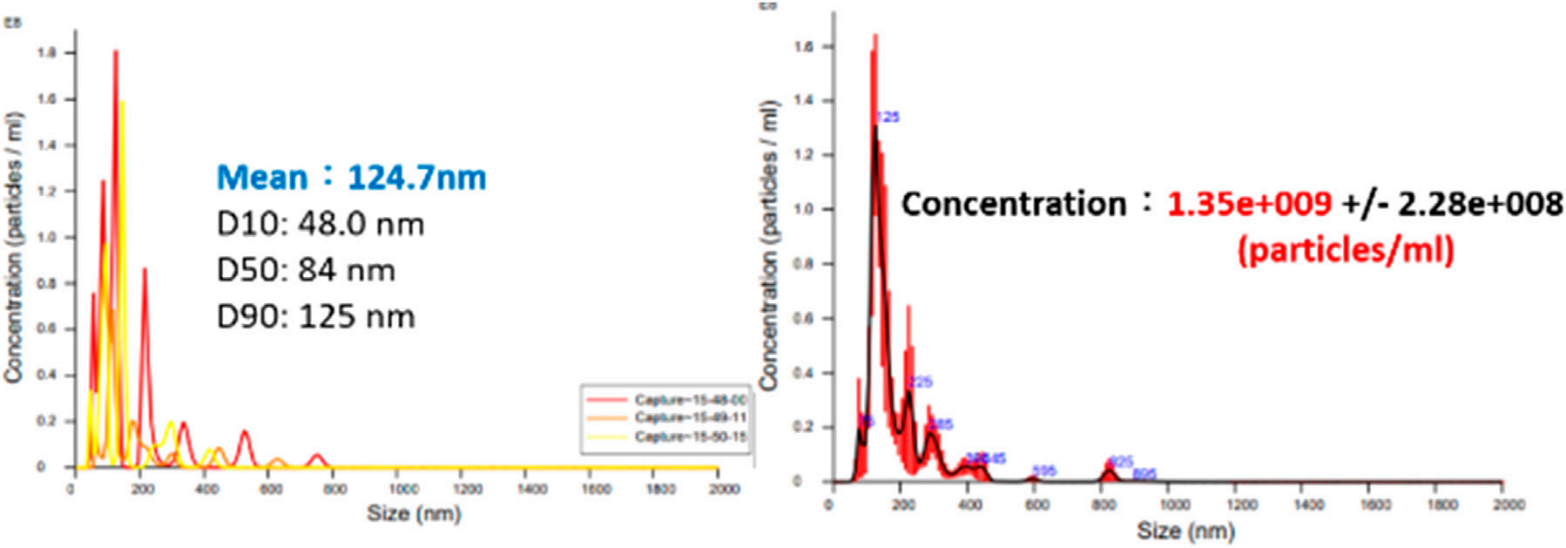
Bubble size and concentration measuring result for nanobubble liquid of porous disc rotary cutting.

Based on the Bernoulli Equation, the characteristics featuring smaller fluid pressure under faster fluid flowing speed are acquired for this device. By triggering the co-axial rotation for hollow mandrel and porous disc, a higher linear cutting speed can be achieved because the radius of the porous disc is bigger than the hollow mandrel. In this way, a low-pressure zone is created surrounding the porous disc in producing the differential pressure through such speed difference; thus, allowing the gas to be sucked by the hollow mandrel, and the gas is then thrown out through the porous disc (first layer bubble refining). As a result, the bubbles are formed on the surface of the porous disc. During the rotating process, the bubbles are cut and refined through the water current and the rotary shearing force of the porous disc (second layer bubble refining). In this way, nanobubbles are produced through the bubble cutting and the refining process, as per Figure 4, Figure 5. Because the entire experiment is conducted in an enclosed chamber, different gases can be conveyed to the chamber in order to replace other gases contained in the nanobubbles whenever required in order to achieve diverse application purposes. Analyzed below are the advantages of porous disc rotary bubble cutting device: 1) Higher changeability through supplying different gases: By feeding different gases, nanobubbles containing different gases can be produced. 2) Higher rotary cutting efficiency: When compared to straight line cutting, the bubbles can be easily formed through the linear speed of rotary bubble cutting. 3) Higher applicability due to structural additivity: Such structures can be added to the mechanism designed with a rotary motion function (e.g., motor or pump) to produce nanobubbles. In addition, the bubble size and size distribution of nanobubbles in water were characterized by nanoparticle tracking analysis (NanoSight LM10HS, United Kingdom) with a 60 mW 405 nm laser scattering source. As indicated in Figure 6, the nanoparticle tracing analyzer proves that the bubble refining device of porous disc rotary cutting can produce nanobubbles that are sized at 124.7 nm of average particle diameter and present in 1.35 x 10^9^ (particles/ml) of concentration.

### Microbubble characteristics experiment

#### Pipe flow resistance reducing characteristics for nanobubbles containing different gases

**FIGURE 7:**
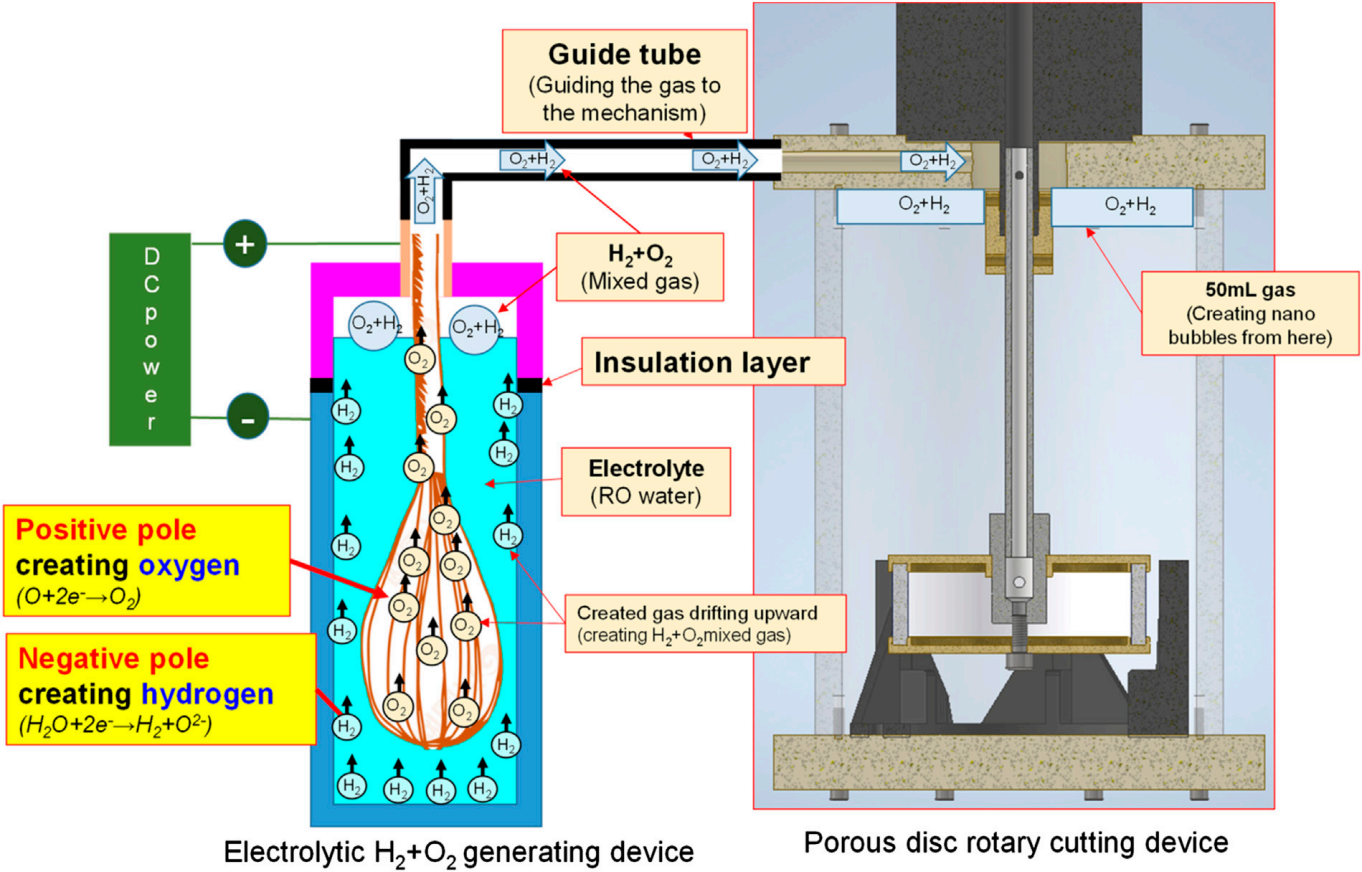
Nano H_2_+O_2_ bubble liquid generating system layout by combining water electrolysis with porous disc device.

**FIGURE 8:**
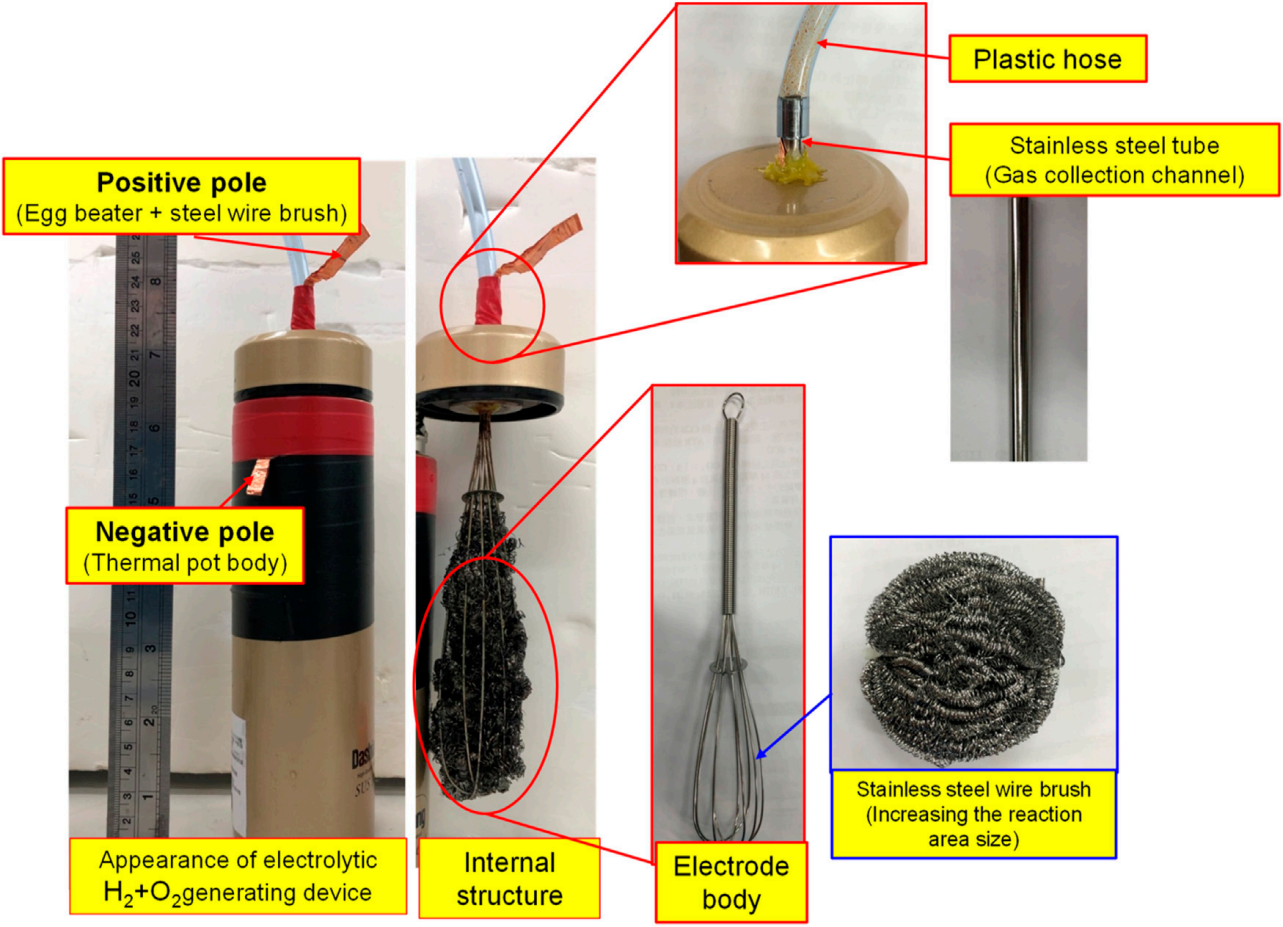
Water electrolysis H_2_+O_2_ gas generating device physical layout.

**FIGURE 9:**
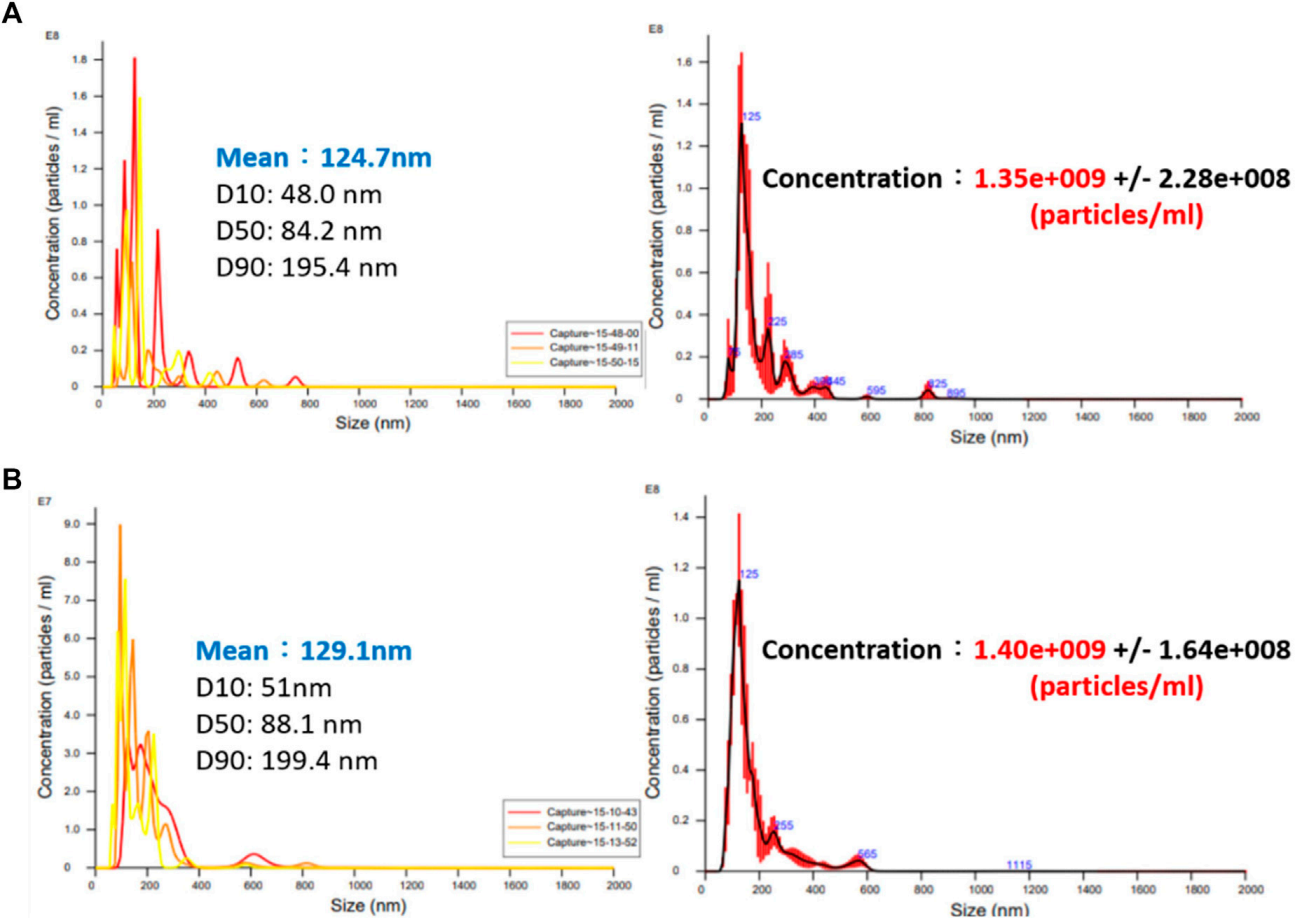
Nano bubble concentration and size produced by different gases **(A)** nano air bubble liquid and **(B)** nano H_2_+O_2_ bubble liquid.

By feeding different gases, the bubble refining device of porous disc rotary cutting developed for this research can produce nanobubbles that are containing different gases. Based on the aforesaid characteristics, the Micro Pipe Flow Titration Test is conducted during the research. The result indicated that different gases will be found when the nanobubbles are present in the same size and concentration. During the experiment, the H_2_+O_2_ gas (mixing H_2_ and O_2_) is produced with the custom-made electrolyte tank, as per the structure indicated in Figure 7, Figure 8. During the experiment, a stainless steel thermos bottle with a bottle body and cap insulated is used as the positive and negative poles. The hole is drilled in the cap and then mounted with stainless steel mesh (for expanding the electrolytic reaction surface area) and RO water is also filled in the bottle for use as the electrolyte. After that, the water electrolysis process is performed by connecting power to the bottle and the cap. As a result, O_2_ is present at the positive pole and H_2_ is present at the negative pole. Under the effect of buoyance, both gases are conglomerated at the upper side, and then highly concentrated H_2_+O_2_ mixed gas is produced. After that, the mixed gas is then guided into the nanobubble generating device, and the nano H_2_+O_2_ bubble liquid is formed. With the aforesaid method, the speed of the nanobubble generating device is set at 3,600 rpm, and then 50 ml air and H_2_+O_2_ mixed gas are guided into 2.65 L of deionized water where the mixed solution is circulated for 3 min to produce nanobubbles. After that, the nano air bubble liquid and the nano H_2_+O_2_ bubble liquid that are containing different gas and that are present at the same size and concentration are obtained, as per Figure 9. It is learned that the concentration and the bubble diameter of the aforesaid liquids are almost the same. The result indicated that after feeding the nanobubbles produced by mixing different gases in the bubble refining mechanism of the porous disc rotary cutting system, the size and concentration will remain unchanged when mixing other types of gas.

#### Electrification characteristics of nanobubbles

**FIGURE 10:**
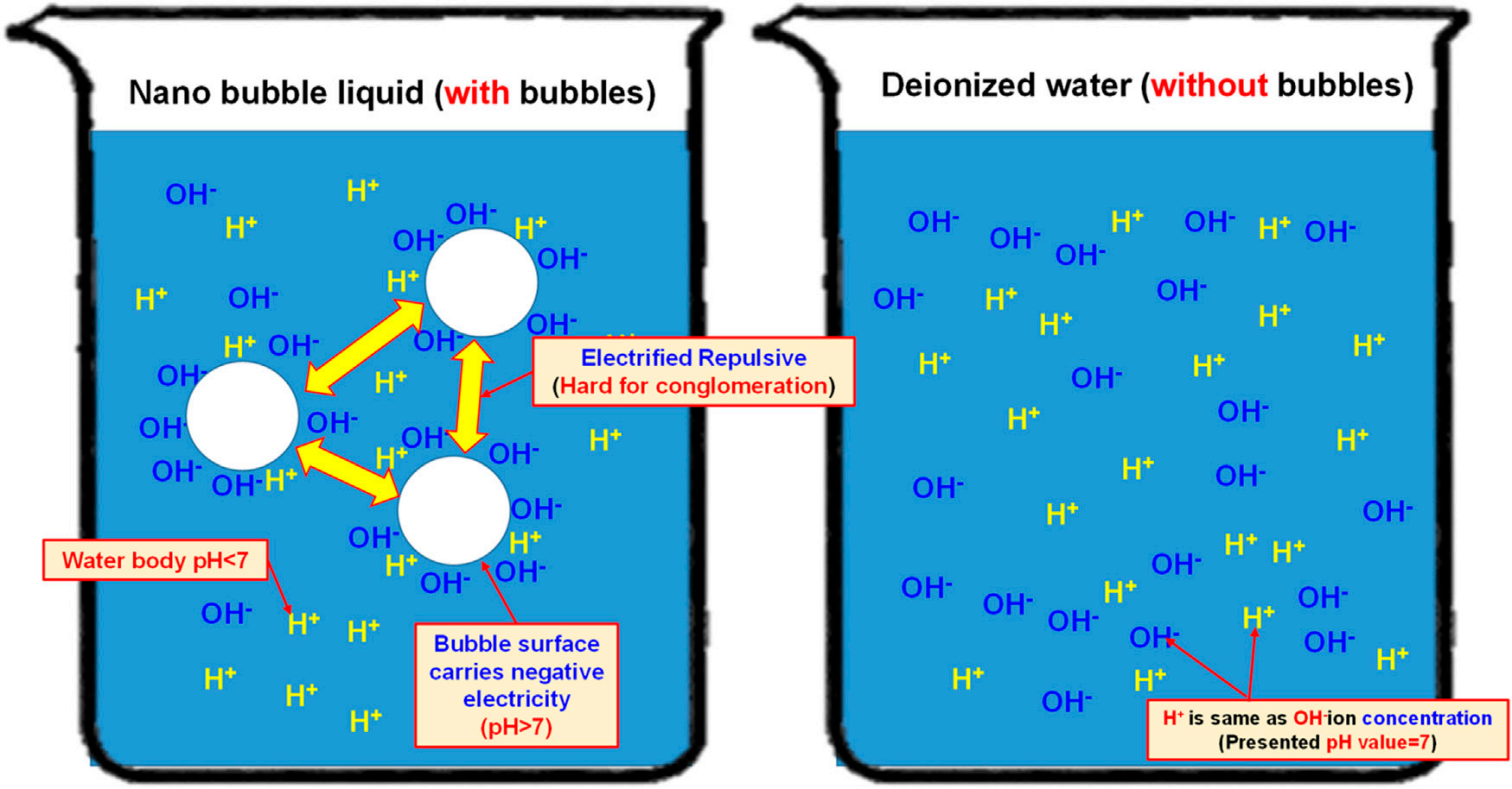
Effect of nanobubbles on H^+^ and OH^−^ ions in the water.

**FIGURE 11:**
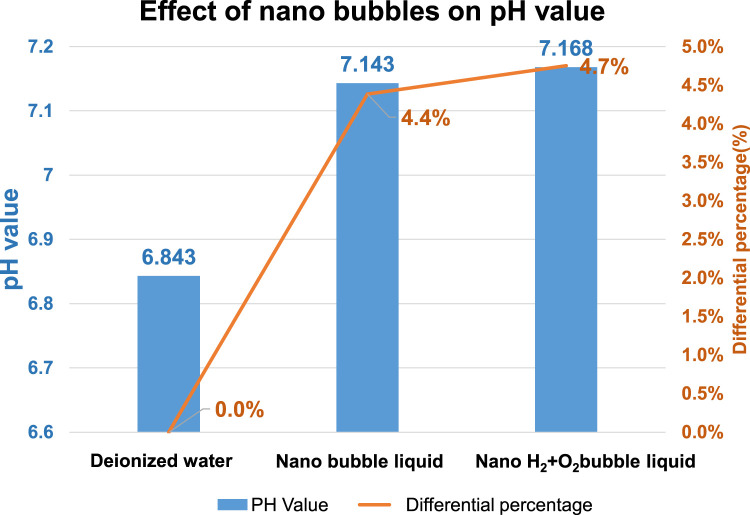
Effect of different types of nanobubbles on pH value.

In 2005, Japanese scholar Masayoshi Takahashi proposed a series of experiments (Kodama et al., 2000). He suggested that the distilled water is presenting the characteristics of an electrified nanobubble surface (Zeta potential) and that the potential surrounding the bubble will drop. When the bubble interface is electrified, the counter ions carrying the opposite charge will be attracted by the surrounding electrostatic and then Double Electric Layer is created. On this basis, it is learned that the surface of the resulting nanobubbles is carrying a negative charge. The pH value is mainly the measured H^+^ and OH^−^ in the water. If the concentration of both becomes 1 × 10-7 mol/L, then the pH value will be 7. If pH is below 7, it means the concentration of H^+^ is higher than that of OH^−^ and the solution is therefore showing the acidifying trend. If pH is higher than 7, it means the concentration of H^+^ is less than that of OH^−^ and the solution is therefore showing the alkalifying trend. It suggested that the higher the concentration of OH^−^ in the water, the bigger the pH value and the stronger the alkalifying trend of the solution. During the experiment, the pH of the nanobubble liquid is also measured to justify the electrifying characteristics of the nanobubbles carrying a negative charge in the water. Based on the description provided for the theoretical mechanism, the quantity of H^+^ and OH^−^ in the water is equivalent (pH = 7). During the nanobubble generating process, the H^+^ and OH^−^ in the water will adsorb onto the bubble surface and the quantity of OH^−^ will be higher than H^+^ (and so the bubble surface is u). In fact, the quantity of H^+^ in the water is already higher than OH^−^ (because OH^−^ is adsorbing onto the bubble surface), and the pH is therefore higher than 7, as per Figure 10. When conducting the test, the porous disc rotary cutting related bubble refining device will be used. Next, the saturated nanobubble solution prepared from the air and the H_2_+O_2_ mixed gas is then poured into the deionized water for measuring the pH value with “twinno PH30 flat-type pH Tester”. Such pH Tester is using the potential to carry out the testing and the measuring. The test is conducted with the composite pH electrode composed by a reference electrode and indicator electrode and then the electrode is soaked in the test solution. When the test solution is presenting varied ion concentration levels, the pH value of such solution is determined according to the potential difference between the reference electrode and the indicator electrode. Indicated in Figure 11 is the measuring result.

**FIGURE 12:**
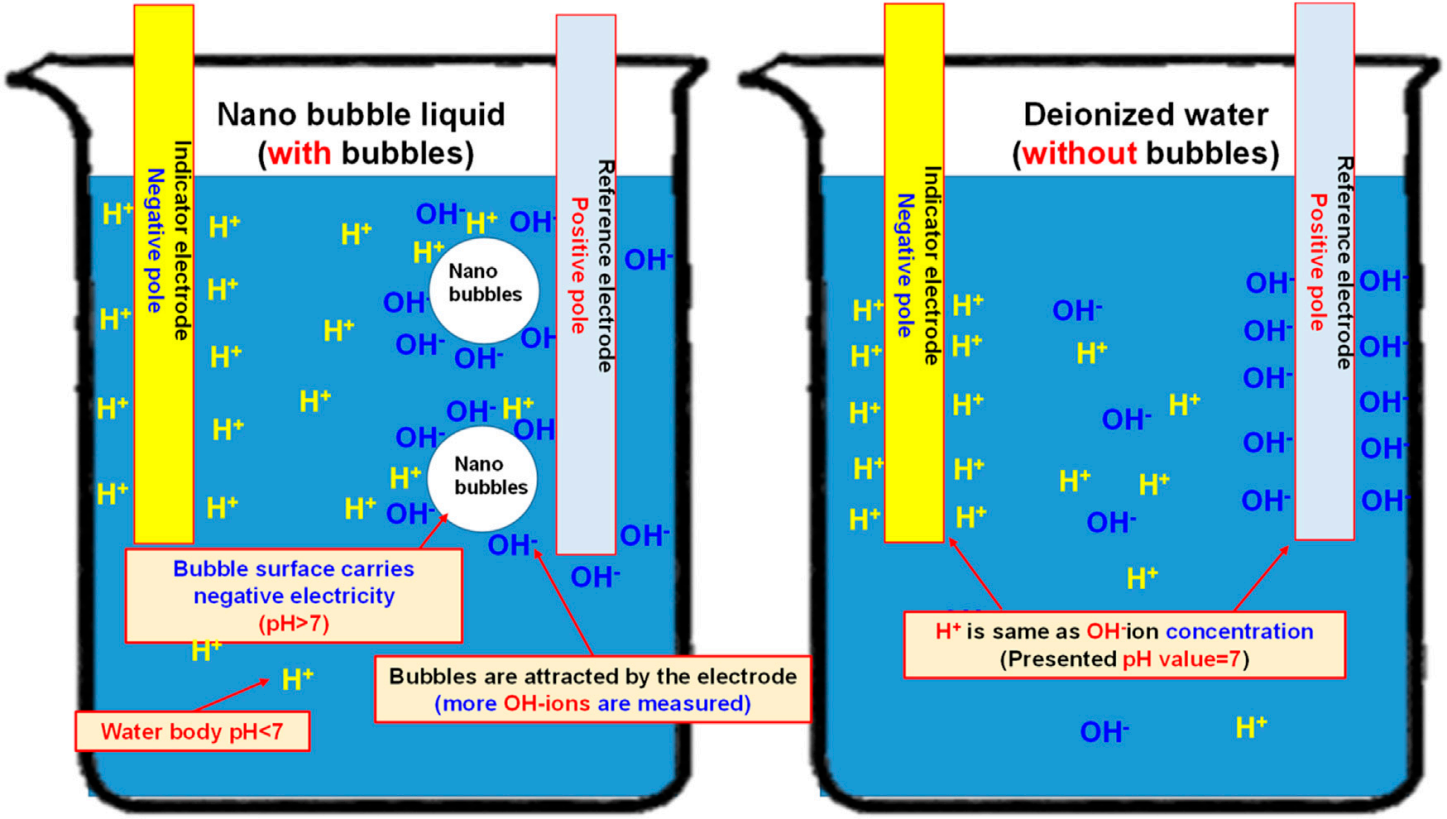
Schematic diagram showing the effect of nanobubbles on the measured pH value (if the bubbles in the water solution carry negative potential, pH is higher than 7; if the water body in the water solution is acidifying, pH is less than 7)

As indicated in Figure 11, the pH value of the deionized water (without bubbles) is present as 6.843 and it has been elevated to 7.143 (an increase of 4.4% as compared to the deionized water-without bubbles) after mixing the nano air bubbles. When mixing the nano H_2_+O_2_ bubbles, it has been elevated to 7.168 (an increase of 4.7% as compared to the deionized water-without bubbles). The result suggests that the pH value will be elevated after mixing the nanobubbles in the water, whether the nano air bubbles or the nano H_2_+O_2_ bubbles. However, the difference between the two is not obvious because it is 0.3%. Such a result should be probably due to the following reasons: As indicated in Figure 12, the pH value of the measured water is calculated according to the potential difference between the indicator electrode and the reference electrode. After mixing the nanobubbles in the water, the OH^−^ ions will conglomerate on the bubble surface (the OH^−^ concentration in the water is dropping). In view that the nanobubbles in the water will adsorb onto the reference electrode (hence, more OH^−^ ions on the surface) during the measuring process, lots of OH^−^ ions will be found on the reference electrode and it has led to the potential difference between indicator electrode and reference electrode. Because more OH^−^ ions are found (pH value will rise), the pH value measured from the water solution is the negative potential representing the pH value (OH^−^>H^+^) on the bubble surface. In fact, the pH value of the water body should be dropping (H^+^>OH^−^). The result indicated that after mixing the bubbles in the deionized water, which type of gas is contained within, the pH value will be slightly increased by 4.4%∼4.7% (pH > 7) when measured with the instrument. It is increased because of the concentration (OH^−^>H^+^) of the electrified ions on the bubble surface. On this basis, the result justified that the surface of nanobubbles is carrying negative potential and the pH of the water body is actually less than 7.

#### Flow rate test for hollow electrode pipe flow jet current

**FIGURE 13:**
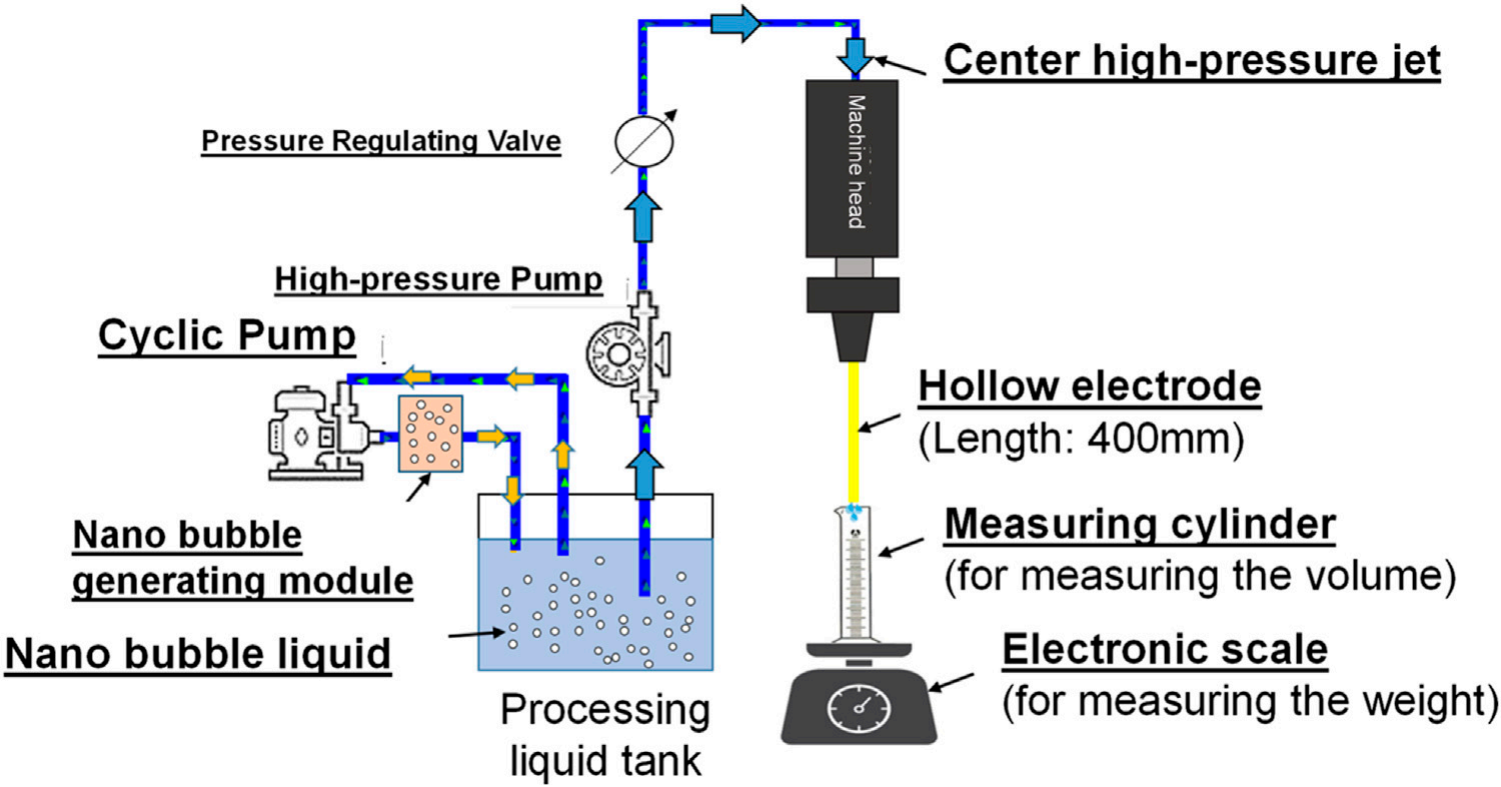
System layout showing the nanobubbles in Pore Discharge Machine jet test.

The jet flow rate test is conducted with “River 350 Pore Discharge Machine (by Ocean Technologies Co., Ltd.). During the test, a high-pressure jet current is formed through the water circulation system of the machine in order to confirm that friction resistance will be reduced for the nanobubbles under a high-pressure environment so as to achieve a higher flow rate, as per Figure 13 below. Based on Hagen-Poiseuille’s equation (Kuhn et al., 2009) $\Delta P=\frac{8\mu LQ}{\pi r^{4}}$ (where, “Δp” means the differential pressure of both ends, “L” means the pipe length, “μ” means dynamic viscosity, “Q” means volumetric flow rate, “r” means channel radius). The result indicated that the pressure loss of the pipe flow is inversely proportional to the radius quartet and that the smaller the pipe diameter, the more obvious in reducing the nanobubble resistance.

**FIGURE 14:**
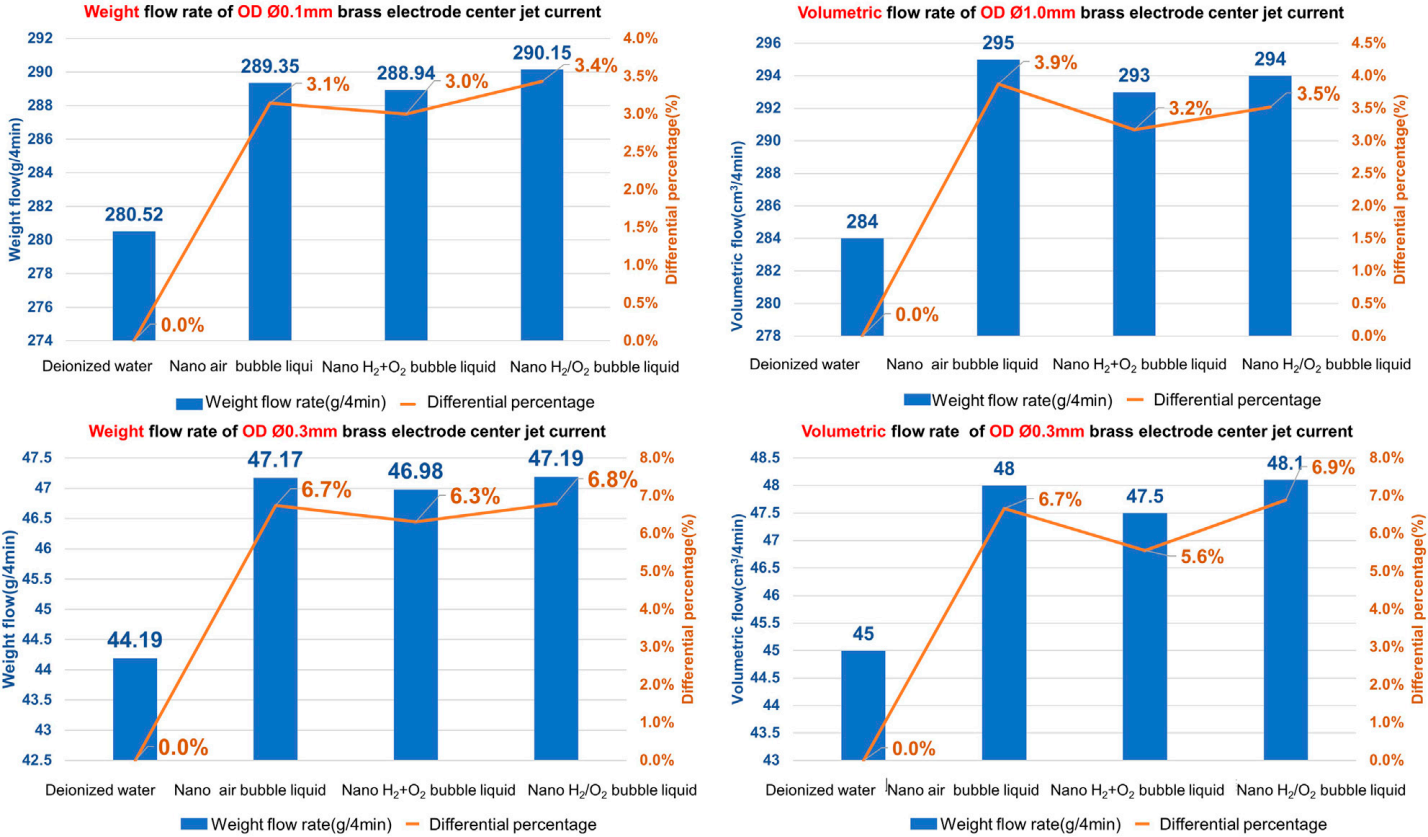
The effect of 3 kinds of nanobubble liquids on high-pressure jet flow.

To conduct the research, the nanobubble generating mechanism is developed to produce the following 3 kinds of nanobubble liquids and they are nano air bubble liquid, nano H_2_+O_2_ bubble liquid (mixing H_2_+O_2_ in the same bubbles), and nano H_2_/O_2_ bubble liquid (mixing H_2_ and O_2_ in the same bubbles respectively). The high-pressure jet current (with pressure rated at 60 kg/cm^2^) is formed from the pore discharge machine head, and it is injected through Ø1.0 mm and Ø0.3 mm (in ID rated at 364 μm and 123 μm), 400 mm long brass electrodes separately. Finally, the glass measuring cylinder and the precision electronic scale are used to record the change of volumetric flow rate and weight flow rate of the jet current being injected within 4 min. Indicated in Figure 14 is the test result.

As indicated in Figure 14, increasing is seen in weight and volume for Ø1.0 mm and Ø0.3 mm hollow electrodes after mixing the bubble liquid with the processing liquid. In terms of weight, the most apparent increase of 6.8% (Ø0.1 mm is increased for 3.4%) is recorded when using nano H_2_/O_2_ bubble liquid in Ø0.3 mm hollow electrode; as for volume, the most apparent increase of 6.9% (Ø0.1 mm is increased for 3.9%) is recorded when using nano H_2_/O_2_ bubble liquid in Ø0.3 mm hollow electrode. As explained in Hagen-Poiseuille’s equation, the smaller the pipe diameter, the more obvious of a fluid pressure loss resulting from the pipe wall friction resistance. As such, the flow rate of Ø0.03 mm is higher than that of Ø1.0 mm.

## Conclusion

During the research, deionized water is used to produce the H_2_/O_2_ gas through the electrolysis process. In addition, high-speed fluid is also applied to cut the gas of the electrolytic module into the nano H_2_/O_2_ bubble liquid for using the fuel processing liquid. In the meantime, the physical porous disc rotary cutting related bubble refining device is also developed through which, different gases are supplied to generate the nanobubbles containing varied gas. When operating under a high-pressure environment (60 kg/cm^2^), the nanobubbles still retain due characteristics in elevating the flow rate by reducing the pipe flow resistance. The test result also suggests that the smaller the pipe diameter, the more obvious of effect allowing nanobubbles to reduce the friction resistance and elevate the flow rate. When applying the aforesaid characteristics in pore discharge and line cutting machining works (narrow seam or micro pipe flow), it will achieve higher cooling and chip removing performance more effectively in order to improve the processing quality and the processing efficiency. In future work, the nanobubble electrification performance related to the pH will be conducted and investigated.

## Data Availability

The original contributions presented in the study are included in the article/Supplementary Material, further inquiries can be directed to the corresponding author.
